# National Ballad Creation Education Under Artificial Intelligence and Big Data

**DOI:** 10.3389/fpsyg.2022.883096

**Published:** 2022-06-21

**Authors:** Xia Liu, Xiao Han, Xiao Lin, Jong Hoon Yang

**Affiliations:** ^1^Art College, Southwest Minzu University, Chengdu, China; ^2^School of Foreign Languages, Xichang University, Xichang, China; ^3^Department of Digital Image, Sang Myung University, Seoul, South Korea; ^4^The International Exchange College, Shandong College of Arts, Jinan, China

**Keywords:** artificial intelligence, big data, ballad creation, teaching work, learning interest

## Abstract

The efficiency of manual ballad creation is low, and the status quo of music creation education still needs to be improved. Therefore, how to upgrade the creative level of students is studied to improve the creative ability of China’s unique ballad culture. The concept of music theory in the process of music creation is explained, and the application of big data in the NetEase cloud music platform is excavated. Besides, the optical music organization (OMR) method based on artificial intelligence (AI) is proposed using a learning method of style imitation. This method is applied to students’ ballad creation education and tested in the school creation curriculum. It is found that the novelty of the ballads created by the system is slightly better than the existing ballads by comparing the ballads created by the machine with those used as imitation templates. In addition, the students’ learning interests and creative achievement are compared through the comparative experiment. The results show that students’ interest in learning has been significantly improved, and their creative performance in oral language has also been enhanced compared with the control class. As a result, this system is considered to be able to be applied in students’ ballad creation courses and provide some basis for AI creation in related fields.

## Introduction

Ballads have always been loved by people. In the past half-century, China has achieved a rapid rise, and its comprehensive strength has increased steadily. The country has gradually gained its position on the world stage. With the advent of the 21st world, the world’s requirements for national strength continue to increase. The prosperity of the country is also inseparable from a new representative of strength - cultural soft power (Zhang and Wu, 2019). In recent years, the competition among major countries is reflected in the cultural soft power excluding the economic dispute. How to convey China’s long-standing cultural history to the world has become a new research direction. Science Technology Engineering Arts Mathematics (STEAM) is a transdisciplinary educational concept that focuses on practice, which is different from the traditional single-subject and book-based education method. STEAM consists of the initials of Science, Technology, Engineering, Mathematics, and Arts (Ozkan and Umdu Topsakal, 2021). The STEAM education concept is proposed by the US government to strengthen the education of K12 in the US in science, technology, engineering, art, and mathematics. The core of the STEAM education concept is to let children complete the projects they are interested in and related to their lives by themselves compared with other pedagogical concepts. This educational concept does not attach importance to educating children to master knowledge but to teaching them to think independently, find, and solve problems. There are various forms of cultural expression. Traditional ways include movies, songs, and books. New ways include large-scale events, competitions, and games (Goyak et al., 2021). However, these channels all have starting requirements, so ballads have become the best way to convey cultural soft power. How to create ballads that can express the unique cultural charm of China is crucial (Lou, 2021).

A cultural ballad can arouse people’s resonance and interest and trigger thinking about Chinese history. Youngblood believed that music could facilitate cultural diversity in different ways (Youngblood, 2019). The premise of creating culturally unique ballads is to strengthen cultural education in relevant aspects. In this cultural education, there is an automatic composition teaching method that can create different types of ballads by imitating various creative styles and themes. Zulić, the first composer to use artificial intelligence (AI), reviewed some possible methods of music education based on AI. Finally, the directions and possibilities of AI in music were introduced (Zulić, 2019). Some studies indicate that AI is gradually playing a role in creation. Sterne and Razlogova analyzed the failure of machine learning in automated audio engineering and summarized the inevitable words of AI. How machine learning could structure or reframe cultural and aesthetic practices was demonstrated to serve digital distribution, identification, and recommendation infrastructure (Sterne and Razlogova, 2021).

The core of the STEAM curriculum is “choice.” In the process of learning and practice, students will find their talents and interests emotionally to realize what they are suitable for learning in the future (Méndez-Porras et al., 2021). The novel teaching method of AI can improve students’ interest in learning, enthusiasm for creation, and performance in song creation (Castelli and Manzoni, 2022). Wu and Song explored the use of social media in entrepreneurship courses from a learner’s perspective. The results revealed that trust, profit, learning, and socialization were the three elements that satisfied the psychology of learning, especially the element of trust, which deserved further study (Wu and Song, 2019). Radoslaw et al. (2018) argued that high scores in vulnerable narcissism and competition predicted weak self-esteem, while high scores in admiration predicted optimal self-esteem. The competition was between vulnerable narcissism and admiration, which supported positioning in the self-importance dimension of the narcissistic spectrum model. Wu et al. (2019) argued that narcissism or spiritual quality influenced educational and practical processes. The above studies all show that improving students’ learning quality and interest is urgent.

Therefore, the optical music organization (OMR) method based on AI is designed in this paper, and it is applied to students’ ballad creation education. An automatic ballad creation system is established using the sample song creation method based on imitation style. The addition of big data enables the system to automatically search for data for ballad creation, providing an automated process for students’ ballad creation courses. Moreover, the reliability of the system is inferred using the student’s creative performance as a judging criterion. The purpose is to provide an automated way for students to take a ballad composition course. The AI ballad creation system supported by big data is an innovative system that does not require human assistance.

## Related Work

STEAM education does not prevent children from making mistakes but encourages them to act. They try different practices, hear different perspectives, and create knowledge that can be applied to real-life through their learning and thinking (Anisimova et al., 2020). STEAM education is a kind of integrated and innovative education, which is based on the matching needs of current education and future social development. The purpose of STEAM is to solve future world problems, fully integrating science, technology, engineering, humanities, mathematics, and other disciplines. It starts by stimulating students’ curiosity to cultivate their continuous interest in learning (Hsiao and Su, 2021). Ballad creation education belongs to the category of art education, and it also implements the concept of art education in STEAM education. At present, the research of experts and scholars in this field has increased. STEAM education is the product of the development of science and technology in modern society. The development of modern society is increasingly dependent on the progress of science and technology. People’s requirements for scientific and technological literacy are getting high. Especially with the emergence of computer technologies such as AI and big data, the current state of education needs the implementation of STEAM education.

Aguilera and Ortiz-Revilla (2021) reviewed the empirical educational interventions based on STEAM to determine its potential to develop student creativity. After a systematic search of papers from the decade 2010-2020, they found 14 teaching interventions in the Web of Science and Scopus databases for analysis during the review process. The analysis results are as follows: (1) STEAM-based interventions have many conflicting forms in theory and practice. (2) Researchers seem to like using Likert-type tests to assess creativity. (3) Both educational methods show evidence of a positive impact on student creativity. The conclusion is that the implementation of STEAM education is conducive to promoting students’ creativity. Immersive technology is rapidly changing the field of education. Augmented reality has shown promise as a resource, especially in STEAM education (Monkeviciene et al., 2020). However, few teachers deploy this new medium directly in the classroom. As a result, only a few elected students benefit from the rich benefits of virtual reality. The curriculum is overloaded, and schools generally lack development resources, so there is no room for experimentation. This situation is further exacerbated by too few educational apps that provide adequate learning content (Jesionkowska et al., 2020). With the development and progress of information technology, AI and machine learning are applied to all fields of life. Among these applications, music has received attention in the past few years. AI-based innovations and technologies are revolutionizing the music industry. It is very convenient for composers to use these techniques to create high-quality music. AI and music are the emerging fields used to generate and manage sound for different media such as the internet and games. The sound effects in the game are good, and it is attractive by implementing AI methods (Yang and Nazir, 2022).

To sum up, the application of AI and big data to music creation is undoubtedly efficient. Ballad creation education can cultivate students’ esthetics and meet the requirements of the current popular STEAM education concept. However, the current research is mostly theoretical and needs research on the actual application of ballad creation. This paper can fill this research gap.

## Materials and Methods

### Research on Music Theory in Ballad Creation

Ballads describe life in the form of lyrics and music, and they are full of deep emotions, experiences, and thoughts. A scale step is a unit that divides intervals between notes in a scale. The essence of the scale step is an independent syllable arranged concerning a specific order, which is divided into basic scale steps and changed scale steps. The basic sound levels are do, re, mi, fa, so, la, and si, which are correspondingly represented by C, D, E, F, G, A, and B (Kearns, 2020). The piano is used to represent distance, the basic scale step is the sound corresponding to the white keys on the keyboard. The changed scale steps are obtained by rising or falling tones. They are classified into ascending scale steps, repeated ascending scale steps, descending scale steps, and repeated descending scale steps (Askerøi, 2021), which are expressed by #, x, b, and bb, respectively. The repeated ascending scale step is one note higher than the basic scale step, and the repeated descending scale step is one note below the basic scale step.

**FIGURE 1:**
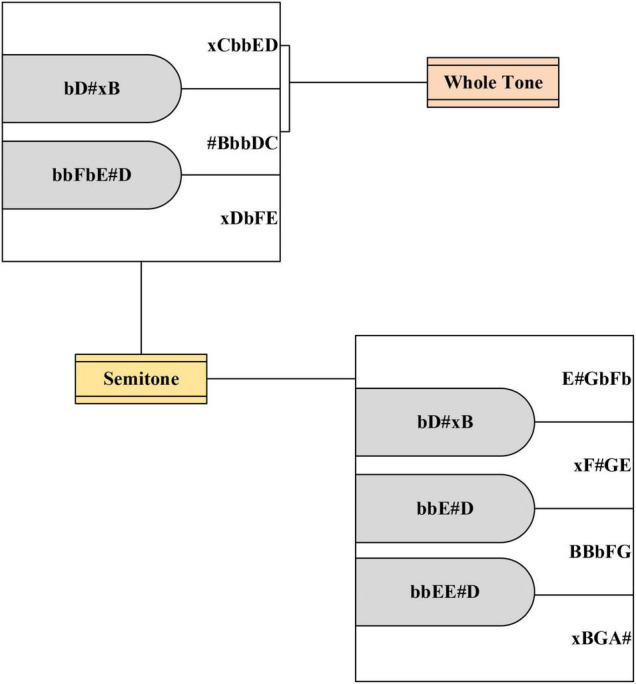
Scale step arrangement method in piano.

The arrangement of several common scale steps in the piano keyboard in the process of ballad creation is shown in Figure 1:

In music, the pitch distance between two notes or scale steps is called an interval (Chen et al., 2019). An octave interval is divided into twelve kinds, each of which is called a semitone. C, D, E, F, G, A, and B correspond to 0, 2, 4, 5, 7, 9, and 11, respectively. Then, the interval can be obtained according to Eq. (1).


(1) I⁢n⁢t⁢e⁢r⁢v⁢a⁢l=F⁢i⁢r⁢s⁢t⁢n⁢o⁢t⁢e-N⁢e⁢x⁢t⁢m⁢o⁢t⁢e+12*(F⁢i⁢r⁢s⁢t⁢u⁢p-N⁢e⁢x⁢t⁢u⁢p)

In Eq. (1), the First note and Next note represent the values from 1 to 12 corresponding to the previous note and the next note. The First up and Next up represent the ascending and descending signs of the preceding and following notes (Strange, 2019), and 12 represents 12 semitones.

As for the calculation method of the scale step, the internal data structure of the file needs to be analyzed. The ordinate of the Do note can be calculated according to different clefs and sharps, and it is called the “DoPlace” value (Sturm et al., 2019). Assuming that there is a high note P in a ballad in the key of X, (r, j) is used to represent the scale step of the entire note. The scale step can be expressed as:


(2) 7⁢r+j=1+D⁢o⁢P⁢l⁢a⁢c⁢e-P

Afterward, the corresponding DoPlace value can be obtained by referring to the number of clefs and sharps in the sample ballad. In addition, the position of the last non-rest note of the song is obtained by the method from back to front, and the ordinate value is recorded as LastNote. According to the above formula, the main note pitch value MainNote can be calculated.


(3) M⁢a⁢i⁢n⁢N⁢o⁢t⁢e=(-1)*(L⁢a⁢s⁢t⁢N⁢o⁢t⁢e-D⁢o⁢P⁢l⁢a⁢c⁢e)+1

### Urban Ballad Creation Based on Internet Big Data

Big data is a new type of data processing mode with strong decision-making and insight capabilities, as well as diversified data assets. It can analyze and filter massive data in the network, and select products and services that are valuable to specific objects (Shen et al., 2020). Data has now appeared in the work of many artists as a focus and creative element but in different ways and roles. Visualizing as a collection is the most common one. As a bright business card in urban culture, landmark buildings are typical representatives of architectural culture. Characteristic buildings can show the personality and characteristics of urban culture, thereby enhancing people’s understanding of urban culture. As the soul of a city, landmarks appear in most urban ballad lyrics. These landmarks can easily resonate with listeners, establish their relationship with the city, and display as a city feature.

The connection between music and society is supported by two forms of communication, namely “natural communication” and “technical communication”. Natural communication is earlier than technical communication. Natural communication is the basis of technical communication, while technical communication is the extension of natural communication. In the era of new media, the ways of transmission of ballads are becoming more and more diverse. Short video application is also a new form for the dissemination of ballads. TikTok is taken as an example. When users upload a video, they choose a soundtrack based on the top song list. These soundtracks have been edited to retain the chorus part, which can form a strong memory in the user’s mind in a short period. There are also related playlists in NetEase Cloud Music, such as “Those Songs People Know But They Don’t Know the Name” and “TikTok Video Background Music Ranking”. “Song of Xi’an People” became popular with the TikTok. It is based on the local urban landscape of Xi’an, such as the Terracotta Warriors, the ancient city wall, and the bowl of wine. This song reflects the typical way of life of Xi’an people and the culture of Xi’an.

**FIGURE 2:**
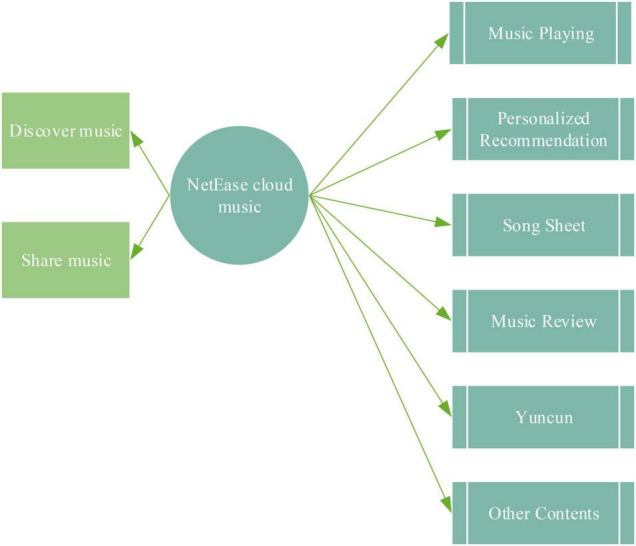
Functional analysis of NetEase Cloud Music.

The Internet media has accelerated the spread of ballads. In this era of rapid consumption, short videos take advantage of the fragmented time of the audience, and each refresh deepens the audience’s impression. There are already 20,000 independent musicians who have accumulated over 400,000 works on NetEase Cloud Music. The functional analysis of big data to help NetEase Cloud Music is revealed in Figure 2:

Musicians have their accounts on NetEase Cloud Music. After fans follow them, new albums are released and sent to fans in the form of private messages as soon as possible. Moreover, in the comment area of the song, some musicians will write down the original intention of the song to deepen the understanding of the audience. This kind of music application combines big data technology to analyze the audience’s preferences and intelligently recommend popular songs that users like to listen to. It can increase the stickiness between users and musicians and is conducive to the dissemination of works by singers. With the help of the Internet and social media, urban ballads can spread the local urban culture by expressing the typical way of life of urban people.

### Recognition and Extraction of Ballad Information Data Based on Artificial Intelligence

**FIGURE 3:**
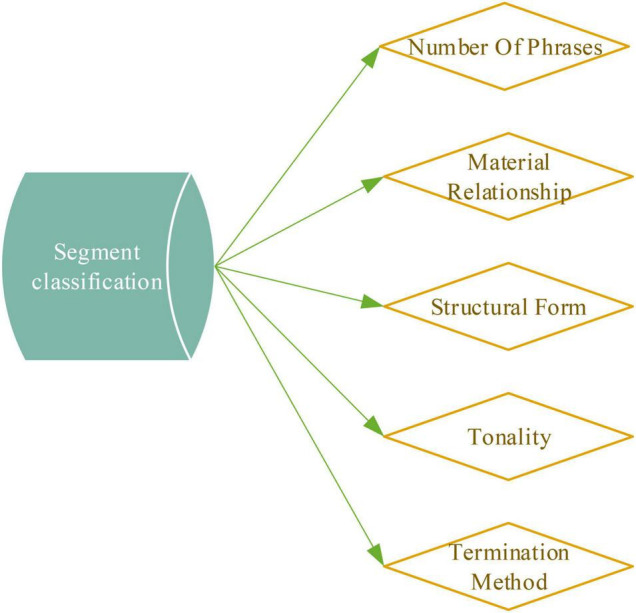
Classification of passages.

Before the model on the ballads selected from the big data is trained, the lyrics in the sample ballads need to be divided into poetic forms. From the division of lyrics to the classification of music, the structure of the ballad is fundamentally determined. The division of exercises in the context of music depends on being able to follow specific division rules (Useche and Hurtado, 2019). Figure 3 displays the division rules.

Model training is required for each ballad sample selected through the big data network. Also, the sentiment types and genres of the sample ballads need to be classified. Ordinary people’s feelings about music are not in terms of rhythm and tone but the emotions expressed in songs, such as excitement, sadness, and joy. People can judge the content of the song by the lyrics. The emotional information of the sample ballads will also affect the music properties corresponding to the lyrics, providing a basis for the automatic creation of ballads. In the training stage, the composition system first needs to divide the expression information of the lyrics (Yu, 2021). The style and characteristics of the ballads are mastered through the whole training process and stored in the corresponding repository. The ballad information that needs to be extracted in the training process includes name, beat, speed, and genre. This information can be directly read and stored in the database. In the subsequent model training process, it is also necessary to obtain the basic information about the music (Baltazar and Saarikallio, 2019), including note assignment sequence, pitch sequence, rhythm note sequence, and decorative note sequence, continuous note sequence, etc.

The structure of a ballad is used to describe the paragraph structure of a piece of music. The structural relationship of the whole music is obtained by arrangement and combination. At present, the development methods of ballad melody are diversified, and it is difficult to compare the number of compositions according to the current one-part, two-part and three-part forms. Therefore, the structure of single music or multi music is used to maximize the efficiency of style learning and structure mining of ballads and express them in a unique structural way.

### Ballad Creation Method Based on Score Recognition

The development of computer science and music technology has led to the extensive research and application of algorithms in computer composition. “Computer-generated art” belongs to algorithmic art. The creator makes the computer automatically generate music or assist him to complete the music creation by writing programs, formulating relevant limiting rules, and other methods. The rise and application boom of technologies such as AI and deep learning have deepened the research and exploration of related theories and technologies. The multi-dimensional integration of technology and music has entered a new historical period. AI composition will also become the main research direction in algorithmic composition in the future.

**FIGURE 4:**
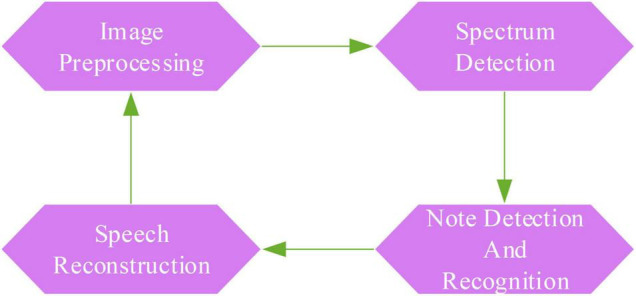
Workflow of OMR system.

The generation of digital musical scores mainly relies on manual input, which not only requires high professional music knowledge of the staff but also requires them to be very familiar with specific music software. Therefore, it is necessary to efficiently and accurately convert musical score visual information into digital information, making the development and optimization of musical score recognition technology an indispensable link in the research background. The MOR system based on AI is presented in this paper. This system is an auxiliary tool for ballad creation, which is convenient for sampling and categorizing musical scores. It can extract musical score data in a unified format and provide guarantees for later development and optimization. The objects of musical score recognition are mainly paper musical scores and digital image musical scores. At present, the main implementation method is to convert digital images through various hardware optical scanning devices to generate binary images. Then, the binary image is further processed. The paper music score is transformed into digital data information through algorithms such as the deletion of staff lines and the positioning of notes. Figure 4 demonstrates the workflow of the OMR system.

**FIGURE 5:**
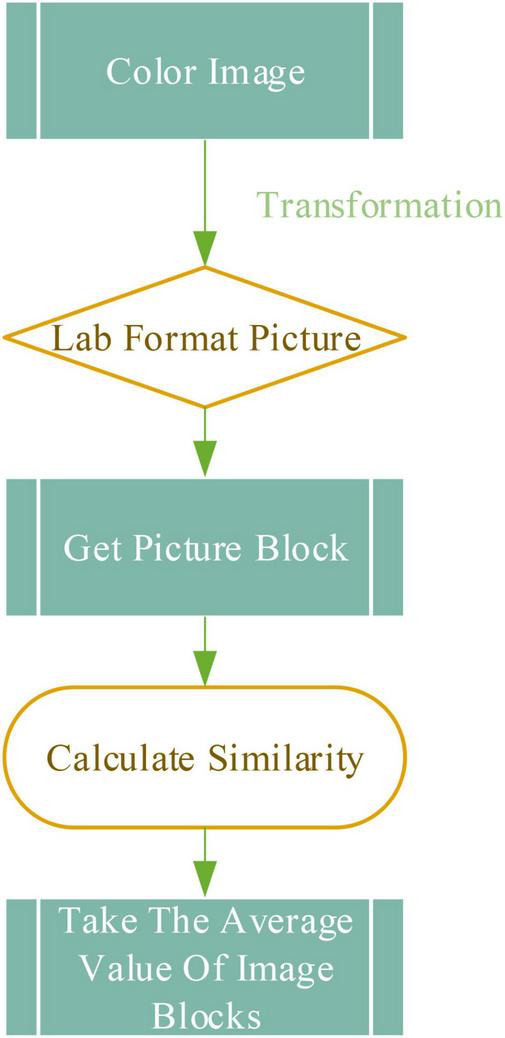
Basic steps of preprocessing.

From Figure 4, this algorithm includes image preprocessing, spectrum detection, note detection and recognition, and speech reconstruction. Image preprocessing first requires image denoising. Image denoising is also called image filtering. Its purpose is to remove some noise points contained in the image through the filter because the existence of these noise points will interfere with the processing of the image. Commonly used filtering algorithms include Gaussian filtering, median filtering, and bilateral filtering. The denoising process is shown in Figure 5.

The image is smooth between adjacent pixels after image denoising. Then, the input image is converted into a binary image, and the entire image contains only two values of 0 and 255. Image binarization processing can not only greatly reduce the amount of image data to improve the processing speed, but also can highlight the contour of the target. The preprocessed pictures can be obtained through the above preprocessing, and then the function of spectrum detection needs to be completed based on these preprocessed pictures.

The thickness and spacing of the spectral lines must first be obtained to complete the task of spectrum detection. Run-Length Encoding is used to calculate the thickness and spacing of the spectral lines. Specifically, a single spectral line is acquired by horizontal mapping. First, it is necessary to obtain how many black pixels are contained in the spectral lines of each row. Then, the black pixels are accumulated into a grayscale histogram, and the input binarized image is mapped onto the grayscale histogram. Each set of spectral lines contains 5 horizontal lines, which correspond to the 5 peak points in the grayscale histogram. In this way, each line in each group of staves can be successfully detected from the score. The first and fifth lines in each set of lines do not contain any notes, so they are deleted. The staff lines of the staff are successfully identified and positioned. Finally, the positioning of the music score identification coordinate system is completed, which provides the necessary support for the subsequent note positioning.

**FIGURE 6:**
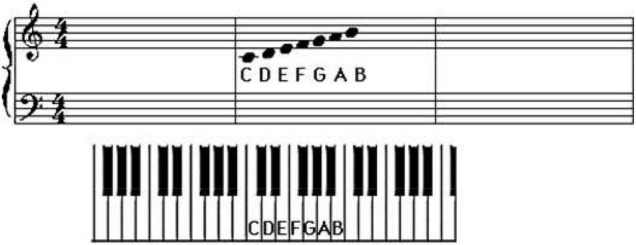
The effect of OMR system note detection and recognition.

The method of template matching is used to realize the task of note detection and recognition based on the above steps. The gray staff is converted into a color image by the function staff_boxes_img, cv2.COLOR_GRAY2RGB. staff_boxes_img indicates the detected staff area, and cv2.COLOR_GRAY2RGB indicates the grayscale of the color method. Each staff is iteratively processed in turn. Each note template is obtained through the function locate_templates(), and template matching operations are performed in different staves. Then, the template matching in the time dimension is carried out. Different notes are matched, and the matched results are displayed in real-time. Figure 6 reveals the specific effect of note detection.

Figure 6 implies that the implemented template matching method can help detect multiple different notes in the staff. The effects of detection and identification meet the requirements for reading basic musical score information, and the accuracy rate is high. The notes in the sheet music picture can be obtained through the above operation. After the notes are accurately acquired, the semantic integrity of the score needs to be guaranteed. The recognized notes need to be organized in a specific order. The score is written by the composer in order from top to bottom and left to right, thereby expressing the time sequence of the music. The semantics between detected notes can be reconstructed according to this principle. The paper scores are electronically stored and saved in a Musical Instrument Digital Interface format to facilitate the establishment of the ballad data set.

### Teaching Process Using the Ballad Creation System Based on Artificial Intelligence

**FIGURE 7:**
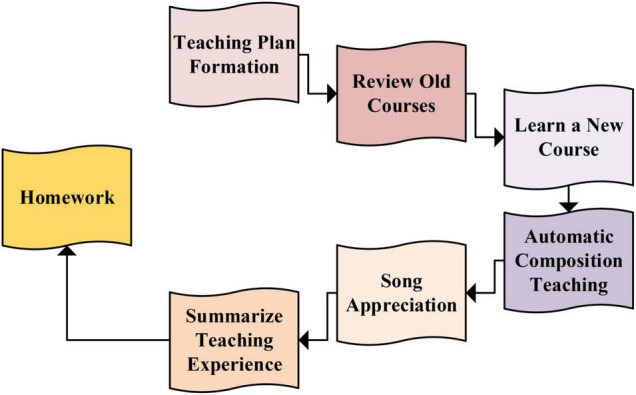
Teaching process using the established ballad creation system.

Figure 7 shows that the teaching process follows the principle of reviewing the old content, learning the new, and teaching the students through automatic composition. With the help of teachers, the teaching process is carried out by the teacher’s guidance, group division, member cooperation, and situational reproduction. The ballad appreciation course is presented by AI, and explanation, appreciation, and practice are combined in the process (Davy et al., 2021).

### Algorithm Structure of Ballad Creation Under Big Data

The used big data control algorithm is an algorithm based on fuzzy cognitive rules. It uses granular computing technology to select semantic concepts from the historical data of the system as the concept nodes of the Fuzzy Cognitive Maps (FCM) model. After that, the global optimization strategy of the Particle Swarm Optimization (PSO) (Sun et al., 2020) is used to solve the weights of the relational degree between concept nodes to form a complete FCM model.

Because research experts found that the description system of FCM has a large difference between the actual values of concepts due to the existence of different entities (Yang et al., 2021), the interval [0, 1] must be used to represent the numerical conversion when constructing the FCM model. If the unified conversion method is used, as shown in equation (4):


(4) $$\text{f}\,\left(\text{x}\right)=\frac{1}{1+{\text{e}}^{-\mathrm{cs}}}$$

It will definitely cause the phenomenon of low comparability of concept nodes, so the parameter σ is formulated for each node, and equation (4) is converted into equation (5):


(5) $$\text{f}\,\left(\text{x}\right)=\frac{1}{1+{\text{e}}^{-\sigma \,\text{s}}}$$

Therefore, for different nodes, the value of σ is also different, and the objective function used is shown in equation (6):


(6) Q=∑i=1c∑k=1Nμ|ikm|Zk-Vi||2

c represents the number of classes, N means the number of samples, $\mu_{i\,k}^{m}$ refers to the membership degree of the sample k in the ith class. m is the control coefficient, *V_i_* is the ith class center, and the performance index of the control C is shown in equation (7):


(7) $$\text{V}=\frac{1}{C\,\left(N-1\right)}\,\sum_{\text{k}=1}^{\text{N}}\sum_{\text{i}=1}^{\text{c}}||{\text{B}}_{\text{i}}^{\left(\text{k}\,1\right)}-{\text{A}}_{\text{i}}^{\left(\text{k}\,1\right)}||$$

$B_{i}^{\left(k\,1\right)}$is the activity level of the actual data *Z_k_* after fuzzy granulation, $A_{i}^{\left(k\,1\right)}$ is the operations of the transition function of applying the FCM model through the state value A_*i*_*^k^* of the node at the previous moment (Weng et al., 2021).

Assuming that the sample set X and the control concept set M are two sets, (M, τ, X) is an adjustment structure, B is contained in X, A is contained in M, the definition is shown in equation (8):


(8) ∧⁢(B)={y|y∈X,τ⁢(x,y)⊇A,∀x∈B}

M is defined as the set of control nodes in the sample set X, then for the control concept A contained in M (Gao et al., 2020), the membership function of the fuzzy concept A is: x∈X.


(9) $$\mu_{\text{A}}\,\left(\text{x}\right)={\text{min}}_{\alpha \in \text{A}}\,\left({\text{L}}_{\alpha }\,\left(\text{x}\right)\right)\in \left[0,1\right]$$

X = {x1,x2,…,xn} is the sample set, F = {f1,f2,…,fn} is the attribute set, M = {m_1,1_,m_1,2_,…,m_1,r1_, m_2,1_, m_2,2_, …, m_2,r2_, …, m_*s,1*_, m_*s,2*_, …, m_*s,rs*_} is the concept set, and the control algorithm of control sample is established, as shown in Table 1.

**TABLE 1:** Algorithms for ballad creation under big data.

1	Start
2	Procedure CN_ FCM Control (W: FCM matrix α: state vector, M:m control variable, N: controlled variable, R: rule, AC: concept aggregate, D: real time data)
3	Begin
4	W = establish Model (R, AC); /*input the rule and concept aggregate to production system, production system make sure the FCM model */
5	α = format Conversion (D); /*production system conversion the data format */
6	begin while (is Target (n)) //estimate the goal of control if to achieve
7	begin while (is End State (α)) /* estimate the state vector if to achieve final state
8 9 10 11 12 13 14	α = FCM Reason (W); //FCM reason operation return the state vector M = extraction (α); //extraction control variable from α m = format Conversion (M); n = incentive (m); /* incentive control variable to real control system and evaluate the return to next time control variable */ N = format Conversion (n); α = (M, N);
15	end
16	end while

## Results

### Comparison Between the Ballad Template and Imitation Template in the Automatic Composition System

Table 2 shows that there are several ballads created by the automatic composition system that adopts the style imitation. The ballads created by the system are compared with the ballads imitated by the system by taking students and teachers as the subjects, and the novelty and pleasant degrees of the ballads are evaluated. The full score is 100 points.

**TABLE 2:** Comparison between creative ballads and imitation ballads.

Number	Emotions expressed	Scores of imitation ballads		Scores of the ballads in the automatic creative system	
		Novelty	Does it sound good	Novelty	Does it sound good
Folk song A	Sadness	64	81	58	36
Folk song B	Love	98	30	96	29
Ballad C	Homesickness	89	41	91	19
Ballad D	Folk custom	98	36	82	20
Military song A	Excitement	72	35	70	31
Military song B	Solemn weight	93	46	82	38
Song A	Freshness	97	39	80	38
Song B	Enjoying the scenery	93	42	79	38
Absolute music A	Violence	78	51	60	48
Absolute music B	Peacefulness	94	70	82	59

Table 2 shows that the scores of the above ten songs show a phenomenon that there is an inverse ratio between the novelty and whether the songs sound good. The ballad is innovative, and it cannot guarantee that it sounds good. On the contrary, the reason for the phenomenon may be because people are used to a kind of music style and are strange to a new style.

After the ballads automatically created by AI are compared with the imitated, it is found that the novelty of the created is better than the imitated, which shows that this automatic composition system can break the fixed style. Whether it sounds good or not is too subjective, and may not well prove the experimental results. However, the novelty and the pleasant degrees of song A and absolute music B in the automatic composition system have higher scores. Therefore, the automatic composition system can be applied in the teaching of ballad creation. For students of music entrepreneurship, big data can become their creative assistant, and can better express the creator’s creative emotions in the ballad creation, to use it as a source of inspiration in the entire creative process. With the assistance of big data and AI, creators can create ballads well and improve their creative efficiency.

### Comparison of Students’ Learning Interest and Their Achievements

**FIGURE 8:**
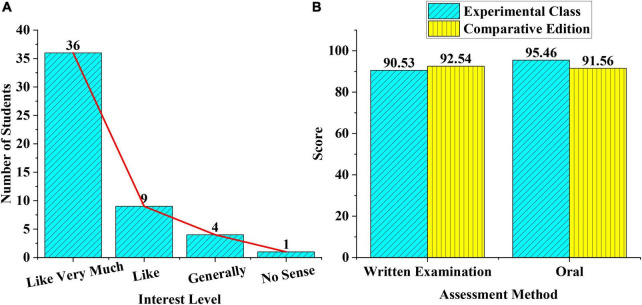
Result statistics **(A)** the class’s interest in the automatic creation ballad system, **(B)** the average scores of written and oral tests between the experimental class and the control class in the mid-term examination.

The automatic creative system is applied to the ballad creation teaching for a class in a school, statistics on whether the middle school students are interested in this teaching method, whether their learning interests are aroused, and their average scores in the midterm examination are made. The scores are compared with the control class in which the students are taught with the traditional system. Figure 8 shows the experimental results of the 50 students in the class.

Figure 8A shows that after the teaching method of the automatic ballad creation system based on AI and big data is used, the learning interest of students in this class is greatly improved. Although 5 students don’t have much enthusiasm for the teaching method, 90% of the students like the creative method. Therefore, the automatic composition system based on AI is feasible.

Figure 8B shows the comparison between the average scores of written and oral tests of the two classes after the midterm examination. In terms of written tests, the average score of the experimental class is about 2.01 points behind those of the control class. In terms of an oral test score, the experimental class is 3.9 points higher than the control class. The reason may be that this automatic ballad composition teaching method based on AI and big data focuses on students’ imitation and creative ability and ignores the basic conceptual knowledge. For ballad creation, the score of the oral test has proved that the teaching method is more effective. AI is slowly changing the way artists think about music. For the industry as a whole, AI tools will enable more efficient, productive, creative, leaner operations, and better-informed decision-making. The industry will continue to grow as more and more record labels add AI software to their rosters, and will soon be a collaborative environment where humans and machines work together to achieve new success.

Therefore, combined with the two experimental results in Figures 8A, B, it is concluded that this teaching method can be applied to the teaching of ballad creation.

## Discussion

The core of Steam education is to discover problems, design methods, solve problems, and verify effects. It does not pursue knowledge points for children to learn to do specific things or answer questions but emphasizes mastering a way of thinking. After students master the method, they can also apply the method to other objects. However, pedagogy studies the field of “education” from an academic perspective. It is not a department to cultivate teachers. It explores the proper state of education from a general direction by studying the nature and purpose of education and the relationship between human growth and education. In addition, it includes not only school education but also education administration, social education, and lifelong education. It also has deep research on human psychology and action. The two educational models of Steam education and pedagogy have their emphases. Songwriting uses big data. The music AI industry is the fusion of music and AI technology. The big data approach to adding AI to the traditional music industry has attracted widespread attention.

As an educational model beyond the traditional, STEAM education narrows the gap between students’ existing knowledge and skills and the knowledge and skills required for their careers and enhances students’ employment competitiveness. Big Data Technology is a high-tech enterprise specializing in music intelligence core algorithm research, chips and software platforms, music intelligence hardware development, and music information services. In the field of music AI, it provides exclusive AI core algorithms, products, services, and solutions for schools, art institutions, consumers, manufacturers, and related application product research and development enterprises. Meanwhile, it is also a pioneer in the field of music AI. When music AI is still in the blank stage of the world, it uses AI deep learning to study music AI from multiple dimensions such as music hearing, vision, and creation. As a result, it promotes the application and popularization of music AI in different fields of the music industry. It takes “creating the future music world” as its corporate tenet and combines the specific needs of consumers in the current music market to launch music AI hardware to meet customers’ needs for all music usage scenarios. It creates the music world of the future, and the music becomes shaping. Big data can help creators reduce workload and integrate their will into ballad creation compared with the previous use of AI as a ballad creation tool.

## Conclusion

Ballad creation culture is the most typical and unique cultural strength of China, and it needs to be displayed specially. Urban ballads about Chinese culture are a means of presentation. Therefore, this paper explores how to improve students’ interest in learning in the teaching of ballad creation. The OMR system supported by AI technology and big data is proposed. The collection of music data through the OMR system is very suitable for the normalized learning of deep learning neural networks. The OMR system not only saves a lot of manual operations but also provides support for the management and expansion of the system. Theoretically, the research on music score recognition technology expands the technical means of recognition and also helps to increase the diversity of image recognition methods. Image recognition technologies in different fields can learn from each other. Technically, the technology-related research on music score recognition can also promote the development of computer intelligence research.

AI creation is applied to the teaching work of ballad creation in a school class. It is found that the novelty of the songs created by this system is better than the imitation of existing songs through experiments. A comparative experiment is conducted in the experimental class and the control class. The result is that the student’s interest in learning is greatly improved, and the performance of the oral composition of songs is better than that of the control class. Therefore, this kind of music score recognition method based on AI and big data platform can be applied to students’ ballad creation teaching work. However, there are some deficiencies in the research. In the first part of the experiment, whether the song is pleasing to the ear is an overly subjective question, and an objective evaluation method should be added to evaluate the results of music creation. In the follow-up work, the songs created will be evaluated by the system in other ways to demonstrate the applicability of this system.

## Data Availability Statement

The raw data supporting the conclusions of this article will be made available by the authors, without undue reservation.

## Ethics Statement

The studies involving human participants were reviewed and approved by Southwest Minzu University Ethics Committee. The patients/participants provided their written informed consent to participate in this study. Written informed consent was obtained from the individual(s) for the publication of any potentially identifiable images or data included in this article.

## Author Contributions

All authors listed have made a substantial, direct, and intellectual contribution to the work, and approved it for publication.

## Conflict of Interest

The authors declare that the research was conducted in the absence of any commercial or financial relationships that could be construed as a potential conflict of interest.

## Publisher’s Note

All claims expressed in this article are solely those of the authors and do not necessarily represent those of their affiliated organizations, or those of the publisher, the editors and the reviewers. Any product that may be evaluated in this article, or claim that may be made by its manufacturer, is not guaranteed or endorsed by the publisher.
